# Intact neural and behavioral correlates of emotion processing and regulation in weight-recovered anorexia nervosa: a combined fMRI and EMA study

**DOI:** 10.1038/s41398-022-01797-1

**Published:** 2022-01-24

**Authors:** Maria Seidel, Sophie Pauligk, Sophia Fürtjes, Joseph A. King, Sophie-Maleen Schlief, Daniel Geisler, Henrik Walter, Thomas Goschke, Stefan Ehrlich

**Affiliations:** 1 Division of Psychological and Social Medicine and Developmental Neurosciences, Faculty of Medicine, Technische Universität Dresden, Dresden, Germany; 2 Division of Mind and Brain Research, Department of Psychiatry and Psychotherapy CCM, Charité Universitätsmedizin Berlin, Berlin, Germany; 3 Department of Psychology, Technische Universität Dresden, Dresden, Germany; 4 Eating Disorder Treatment and Research Center, Department of Child and Adolescent Psychiatry, Faculty of Medicine, Technische Universität Dresden, Dresden, Germany

**Keywords:** Psychiatric disorders, Neuroscience, Human behaviour

## Abstract

Altered emotion processing and regulation mechanisms play a key role in eating disorders. We recently reported increased fMRI responses in brain regions involved in emotion processing (amygdala, dorsolateral prefrontal cortex) in acutely underweight anorexia nervosa (AN) patients while passively viewing negatively valenced images. We also showed that patients’ ability to downregulate activity elicited by positively valenced pictures in a brain region involved in reward processing (ventral striatum) was predictive of worse outcomes (increased rumination and negative affect). The current study tries to answer the question of whether these alterations are only state effects associated with undernutrition or whether they constitute a trait characteristic of the disorder that persists after recovery. Forty-one individuals that were weight-recovered from AN (recAN) and 41 age-matched healthy controls (HC) completed an established emotion regulation paradigm using negatively and positively valenced visual stimuli. We assessed behavioral (arousal) and fMRI measures (activity in the amygdala, ventral striatum, and dorsolateral prefrontal cortex) during emotion processing and regulation. Additionally, measures of disorder-relevant rumination and affect were collected several times daily for 2 weeks after scanning via ecological momentary assessment. In contrast to our previous findings in acute AN patients, recAN showed no significant alterations either on a behavioral or neural level. Further, there were no associations between fMRI responses and post-scan momentary measures of rumination and affect. Together, these results suggest that neural responses to emotionally valenced stimuli as well as relationships with everyday rumination and affect likely reflect state-related alterations in AN that improve following successful weight-recovery.

## Introduction

Contemporary models of eating disorders (ED) including anorexia nervosa (AN) propose altered emotion processing and emotion regulation mechanisms to play a key role in the development and maintenance of the disorder [1–4]. Acutely underweight AN patients (acAN) have been reported to have difficulties in general emotional processing [5–8]. In addition, impairments in emotion regulation have also been identified, for example, more frequent use of emotion regulation strategies that are considered maladaptive, such as suppression or rumination [9, 10]. Most research on emotion processing and regulation in AN to date has been based on self-report [3]. However, notions of discrepancies between self-reported emotional and physiological reactivity to emotional stimuli [11, 12], as well as high levels of alexithymia in AN [13] warrant a cautious interpretation of these results.

Functional neuroimaging studies generally support the aforementioned behavioral findings suggestive of altered processing of emotions in acAN individuals [14, 15] and show differences in neural responses in visual, limbic as well as frontal brain regions [16–19]. We recently reported increased responses of the right amygdala as well as the bilateral dorsolateral prefrontal cortex (dlPFC) in acAN patients while passively viewing negatively valenced images [20]. Results were interpreted as an increased emotional reactivity to negative stimuli (amygdala), possibly as a result of depleted resources due to continued attempts at regulating (increased dlPFC) negative emotions. However, contrary to previous findings from questionnaire and self-report data [3], we did not find any differences between acAN and healthy control participants (HC) in the neural regulation of either negative or positive emotions and corresponding arousal ratings [20, 21] during an established task which required emotional reappraisal via distancing [22]. This was found despite the fact that patients generally reported less (trait) reappraisal as assessed with the Emotion Regulation Questionnaire (ERQ [23]. Yet, we also uncovered that patients’ ability to downregulate activity in a reward-related brain region (ventral striatum [VS] [24]) during the regulation of positively valenced stimuli was predictive of increased rumination and negative affect, (assessed several times daily via ecological momentary assessment (EMA) for 14 days after the scanning session in real life), and, more importantly, worse treatment outcome (weight gain). Previous research has emphasized the costs and negative consequences of excessive self-control within a limited capacity model [25, 26]. Consequently, it has been suggested that cognitive processes such as emotion regulation or emotional avoidance might exhaust control resources [21,27], which subsequently has negative consequences (costs) by facilitating the development of ED-related symptoms such as heightened negative affect and ruminative thinking.

Most studies of emotion regulation in AN, including our own previous investigation of neural processing and regulation of emotions [20, 21], focused on acAN and results may thus be partially biased by consequences of insufficient food intake and the cachectic state with all its metabolic and endocrine consequences [28–30]. Therefore, forthcoming research needs to address whether group differences found between acAN and healthy individuals are due to state factors (e.g., the undernourished state) or whether they present a trait marker, potentially influencing the development and maintenance of the disorder. Studying weight-recovered individuals with a history of AN (recAN) could help answer this question. Previous investigations into self-reported aspects of emotion processing and regulation in recAN have yielded heterogeneous results [31–34] while investigations using functional MRI are scarce and have not included an explicit regulation condition [35, 36].

The aim of the current study, therefore, was to investigate possible alterations in the processing of negative emotional stimuli as well as negative consequences of regulating positive emotional stimuli (as previously found in our sample of adolescent acAN) after weight-recovery. To this end, we used the same emotion regulation task [20, 21] in recAN to test for persistent alterations in the neural correlates of emotional processing as well as regulation (distancing) as a voluntary strategy to reduce arousal. Using subjective (arousal ratings) and objective (fMRI) data, we were interested not only in whether former patients (recAN) would continue to show increased reactivity to negative stimuli but also display increased negative consequences of regulating positive emotions. As in our previous study in the acAN sample, this was assessed by combining measures of the fMRI emotion regulation task with data from an EMA study assessing disorder-specific rumination as well as affect in real life.

## Method

### Participants

Data were collected from 41 recAN and a total of 50 HC. HC were recruited with the goal to match the samples for age. To optimize comparisons between recAN and HC we implemented a pairwise matching algorithm [37], resulting in a sample of 82 female volunteers: 41 recAN (15.4–29 years) and 41 female HCs (15.5–29.7 years). This procedure resulted in a maximum difference of 0.8 years between matched pairs. There was no overlap in participants between the acAN patients in our previous studies [20, 21] and the current sample of weight-recovered participants. All recAN participants had an AN diagnosis in the past according to DSM-IV obtained with the expert version of the Structured Interview for anorexia and bulimia nervosa (SIAB-EX [38]). To be considered “weight-recovered”, recAN subjects had to (1) maintain a BMI (kg/m^2^) >18.5 (if older than 18 years) or above the 10th age percentile (if younger than 18 years) for at least 6 months; (2) menstruate; and (3) have not binged, purged, or engaged in restrictive eating patterns during at least 6 months before the study. Further sample descriptives are provided in Table 1. HC participants had to be of normal weight, eumenorrhoeic, and without any history of psychiatric disorder. Exclusion criteria for both groups and possible confounding variables, e.g., the use of psychotropic medication and medical comorbidities, were obtained using the SIAB-EX [38], our own semi-structured research interview, and from medical records. For additional exclusion criteria see supplementary material 1.1.

**Table 1:** Descriptive statistics, results of group comparisons using independent samples *T*-tests, displaying mean and standard deviation.

Descriptive Statistics	(recAN/HC)	recAN		HC		
		Mean	SD	Mean	SD	
**Age**	41/41	22.10	3.67	22.05	3.68	
**BMI**	41/41	20.65	1.60	21.93	2.02	**
**BMI-SDS**	41/41	−0.53	0.56	−0.11	0.56	**
**Duration of Recovery**	41/41	58.72	54.97			
**BDI-II**^**a**^	41/41	10.15	10.00	4.40	4.66	*
**EDI-total**	41/40	178.93	52.36	139.29	25.00	*
**ERQ-Reappraisal**	41/41	48.20	8.99	50.98	10.12	
**ERQ-Suppression**^**a**^	41/41	51.07	9.24	49.17	8.70	

Duration of recovery is given in months, range: 9–270 months (only *n* = 2 < 12 months). Previous AN diagnoses of recovered individuals included *n* = 34 of the restrictive subtype and *n* = 8 of the binge/purge subtype.

*RecAN* recovered Anorexia nervosa, *HC* healthy control, *BMI* body-mass index, *BMI-SDS* BMI standard deviation score, *EDI-2-total* eating disorder inventory 2 total score, *BDI-II* Beck depression inventory, *SD* standard deviation.

***p < 0.001, **p < 0.01, *p < 0.05.

^a^As normal distribution of variables was not given group comparisons were repeated with Mann-Whitney U-tests. Results did not change (supplementary material Table S1).

This study was approved by the local institutional ethics review board, and all participants (and their legal guardians if underage) gave written informed consent.

### Clinical measures

To complement the information obtained with the clinical interviews, we assessed ED-specific psychopathology using the Eating Disorder Inventory (EDI-2) [39] and depressive symptoms using the Beck Depression Inventory (BDI-II) [40]. For trait use of the emotion regulation strategies reappraisal and suppression, we used the Emotion Regulation Questionnaire (ERQ) [41]. BMI and BMI standard deviation scores corrected for gender and age (BMI-SDS) [42] were collected on the day of scanning.

### Emotion regulation task

During the task, participants were asked to either passively view sets of negative, positive, and neutral pictures or to actively downregulate any emotions arising in response to the negative and positive pictures [43, 44]. During the “view” condition participants were instructed to simply look at the picture without modulating any associated feelings, without looking away or distracting themselves in any way. During the regulation condition, they were told to downregulate any elicited feeling via the reappraisal strategy “distancing”. After each picture presentation (6 s) participants were asked to rate their emotional arousal. Participants first completed a practice session. The main task consisted of 100 trials (20 per condition) which were presented in pseudorandomized order with each condition constrained to not occur more than twice in a row, while the assignment of stimuli to either the “view” or “distance” condition was randomized for each participant. The fMRI measurement lasted for ~23 min. See supplementary material 1.2 or Seidel et al. [20, 21] for more details about the task and procedure.

### Ecological momentary assessment

Rumination about AN-related content (food/weight) was assessed via two items adapted from the SIAB-EX interview i.e., “How much have you been thinking about food/calories/cooking?” and “How much have you been thinking about your weight/shape?”. Responses were ranging from “not at all” to “a lot”.

An adapted version of the Multidimensional Mood Questionnaire (MDMQ) [45] recommended to use in EMA research [46] assessed calmness and valence of affect with two bipolar items each. Higher scores indicated more positive affect and more calmness.

The app-based questionnaire was designed via an online platform (MovisensXS, Karlsruhe, Germany), which also managed data collection and immediate server upload. EMA sampling started the day after the fMRI scan and lasted for a period of 14 days. Data collection occurred via the signal-contingent assessment method: Alarms occurred at six semi-random times during a 14 h period that was adapted for each individual to suit different daily routines. For further details of the EMA study design and procedure see Seidel et al. [10] or supplementary material 1.3.

### Functional image acquisition and processing

Images were acquired between 8 and 9 a.m. following an overnight fast using standard sequences with a 3 T whole-body MRI scanner (TRIO, Siemens) equipped with a standard head coil. Functional and structural images were processed with SPM8 (www.fil.ion.ucl.ac.uk/spm) within the Nipype framework (http://nipy.sourceforge.net/nipype/37) following standard procedures, an artifact detection tool and DARTEL (for details of fMRI acquisition and processing, see supplementary material 1.4).

On the single participant level a general linear model (GLM) was fit to model the brain activation in response to each of the five conditions (neutral; positive watch or distance; negative watch or distance). We modeled the picture as a boxcar function with a duration of 6 s and the subsequent rating as stick-function (zero duration). Additional regressors included six motion parameters and one regressor for each motion or intensity outlier volume as nuisance regressors of no interest. All events were modeled using a canonical hemodynamic response function.

### Statistical analysis

Analysis strategies for task-related effects of negative and positive emotional processing and regulation, as well as EMA data were based on our previous analyses in an independent sample of acAN as outlined in the introduction [20, 21]. Therefore, primary analyses comprised all analyses including variables that were found to be different between acAN and HC in our previous studies [20, 21]. Secondary analyses included group differences in the main effects of tasks and associations with clinical and demographic variables.

#### Clinical and behavioral task-based data

Analyses included independent samples *t*-tests or Mann–Whitney *U*-tests (if the normal distribution was not given), to compare clinical and questionnaire data between groups using SPSS 26. Secondary analyses were comprised of a 5 × 2 repeated measures ANOVA, to test for potential group differences across the conditions of the emotion regulation task (neutral, negative watch, negative distance, positive watch, and positive distance) in the arousal ratings.

#### Functional MRI

The first part of the primary analysis included the investigation of group differences in the contrast negative watch>neutral (as in acAN [20]). Regions of interests (ROIs) were defined by AAL regions with significant group differences between acAN and HC in this contrast, i.e., right amygdala and right and left dlPFC, as implemented in the WFU PickAtlas toolbox for SPM [47, 48]. Of note, as no significant group differences had been found in the left amygdala all contrasts for the ROI were investigated as part of the secondary analysis, see below. To calculate group differences between recAN and HC we applied second level independent samples *t*-tests within the respective ROI. To control for false positives, familywise error correction was performed using 3DClustSim (https://afni.nimh.nih.gov/, version from 3 July 2017). Specifically, the program was used to run 10,000 Monte Carlo simulations to estimate the cluster size above which the false positive probability is below a given α-level (α = 0.05) for a given voxel-wise *p* value, which was set at 0.001. At this voxel-wise threshold (two-sided), clusters with more than six voxels for the right amygdala and 33 voxels for the left and right dlPFC each, corresponding to a combined threshold of *p* < .05 [familywise error (FWE) corrected]. In case of nonsignificant group differences in the data derived from neuroimaging, we used the JASP software to calculate Bayesian independent *t*-tests to verify the absence of any group effects [49]. In order to do so, we averaged indices of activation (β estimates) which were extracted from the aforementioned ROIs using the MarsBaR toolbox [50]. A Bayes factor (BF01) >3 represents moderate and BF01 >10 strong evidence for the null hypotheses (no group differences).

The second part of the primary analysis investigated group differences in the association between neural indices of positive emotion regulation with post-scan measures of affect and rumination in real life (EMA data; as found in acAN [21]). Mirroring the analyses strategy in our acAN study, we calculated a positive neural emotion regulation score using extracted betas from the VS (defined by AAL regions in which we had found significant group differences between acAN and HC). The score was calculated by subtracting the extracted betas within the VS during the condition positive distance from the positive watch. Subsequently, the higher the positive neural regulation score, the more neural activity was reduced during the regulation condition as compared to the watch condition. For further analyses investigating associations between the positive neural regulation score and EMA data refer to the hierarchical linear model section.

Secondary analyses included investigating group differences during watching negative pictures (negative watch>neutral (left amygdala)), regulation of negative pictures (contrasts: negative watch<negative distance (bilateral amygdala), negative watch>negative distance (bilateral dlPFC)) as well as viewing and regulation of positive pictures (positive watch>neutral (VS), positive watch>positive distance (VS), positive watch<positive distance (bilateral dlPFC)) via independent *t*-tests. Again, clusters with more than six voxels for the right/left amygdala and 33 voxels for the left and right dlPFC each, corresponding to a combined threshold of *p* < .05 [familywise error (FWE) corrected. Secondary analyses also included whole-brain analyses as well as investigating associations between imaging and behavioral and clinical data (supplementary material 1.5.1).

#### Hierarchical linear models

As not all participants had provided EMA data, HLM analyses were calculated on a subsample of 65 (recAN = 30, HC = 35) individuals. EMA data of affective variables and rumination of a largely overlapping (70.73%) sample of weight-recovered patients has been published previously [51]. As the research design of the EMA data yields nested data, primary analyses included conducting hierarchical linear models (HLM 8) [52]. As in our previous study we set up four different models to examine the extent to which positive neural regulation was able to predict disorder-relevant rumination (food: model a; weight: model b), the valence of effect (model c), and calmness (model d) in the 14 days following the scan [21]. These models took into account that the dataset was organized within three different levels with single observations (Level 1) nested within days (Level 2) which were nested within participants (Level 3). The same statistical approach was used for all models. In model a–d we allowed for random intercepts and included time (indicating time of day as a continuous variable from 1 to 6) on level 1 and day of study (1 to 14) on level 2. On level 3, the person level, we included three predictors. The first diagnostic group was inserted, coded as 1(recAN) and −1(HC). Second, we entered the mean-centered positive neural regulation score (positive watch - positive distance) of the extracted betas from the VS (as described above), and thirdly we included an interaction term of this regulation score with a diagnostic group. For supplementary analysis we also looked at effects of negative emotion regulation on rumination and negative affect by conducting separate HLMs that included extracted data from the amygdala during negative regulation as a predictor instead of the VS (for details see supplementary material 1.5.2).

## Results

### Clinical variables and behavioral task-based data

We did not find any differences between recAN and HC in age or IQ, but BMI was significantly lower and ED symptoms (EDI-2), as well as depression scores (BDI-II), were still elevated (Table 1). As indicated by ERQ subscale scores, recAN did not show any differences in the general use of the emotion regulation strategies reappraisal and suppression. As normal distribution was not given for BDI-II and ERQ-suppression scales, group comparisons were repeated using Mann–Whitney *U*-tests. The significance of the results was identical (supplementary material Table S1). Secondary analyses of arousal ratings acquired during the fMRI task revealed no group differences in the five conditions (see supplementary material 2.1).

### Functional MRI

Contrary to our previous findings in acAN [20], primary analyses revealed no group differences in neural processing of negative stimuli in the right amygdala and the bilateral dlPFC between recAN and HC (contrast: negative watch>neutral). Post hoc analysis using Bayesian independent samples *t*-tests showed moderate evidence in favor of the null hypothesis (BF_01_ right Amygdala = 4.19, BF_01_ right dlPFC = 4.32, BF_01_ left dlPFC = 4.32) confirming the absence of group differences between recAN and HC in these ROIs.

Results of secondary analyses investigating group differences in effects of the task as well as analysis of associations between neuroimaging and questionnaire data are presented in the supplementary material 2.2 (fMRI task-based findings) and 2.3 (associations between behavioral and neuroimaging findings). In short, there were no group differences in the neural activity in any of the contrasts in any ROI (negative watch>neutral left amygdala, watch>negative distance in the bilateral amygdala and positive watch>neutral, positive watch>positive distance in the VS and positive watch<positive distance in the bilateral dlPFC). The downregulation of amygdala activity in the negative watch>negative distance contrast was mirrored by the downregulation of subjective arousal (supplementary Table S2).

### Hierarchical linear models for EMA data

Applying the same analytic approach previously employed in our acAN sample [21], we tested the effect of the positive neural regulation score based on VS activity (and its interaction with the group factor) on rumination about weight (model a), rumination about food (model b), affect (model c), and calmness (model d) based on a total of 4361 data points.

The recAN group showed elevated rumination about weight, more negative affect, and less calmness than HC (for more details on EMA data see supplementary material Table S3 or Fürtjes et al. [51]). However, contrary to our previous findings in acAN, there was neither the main effect of positive neural regulation nor a significant positive neural regulation x group interaction in any of the models. Evidently, neural downregulation of the VS did not predict subsequent rumination about weight, rumination about food, negative affect, or calmness levels in either group (see supplementary material Table S3. Supplementary analysis investigating effects of negative emotion regulation on EMA data did not show any significant effects (supplementary Tables S4 and S5).

## Discussion

Building on our previous findings in acAN patients [20, 21], the current analysis sought to test for persistent alterations in the processing of negatively valenced emotional stimuli and the real-life consequences of regulation of positive emotions after weight-recovery from AN. In contrast to our findings in acAN [20], primary analyses revealed no group differences between recAN and HC in the amygdala and dlPFC during passive viewing of negatively valenced images. Further, there was no significant association between the voluntary downregulation of neural activity in the VS in response to positively valenced stimuli and either negative effect or disorder-related rumination in real life during the 2 weeks after the scanning session (although negative affect and rumination were still elevated in recAN). In line with that, questionnaire data confirmed that recAN use trait reappraisal to the same degree as HC, suggestive of a normalization process after recovery. Secondary analyses showed that recAN also did not display any difficulties downregulating negative and positive emotions when instructed to use distancing, as measured using arousal ratings and neural activity in the amygdala, the VS, as well as dlPFC. When considered in light of our previous findings in acAN, the data at hand suggest that alterations of emotional processing and increased negative real-life consequences of regulation do not persist following long-term weight-recovery and are thus more likely to constitute correlates of the underweight state than trait factors of the disorder.

In contrast to the increased amygdala and dlPFC activation during passive viewing of negative stimuli we previously observed in acAN [20], we found no evidence of any such neural alterations in recAN. The amygdala is a complex structure important for detecting potentially threatening and fearful stimuli [53]. As such, it is highly responsive towards all emotionally valenced stimuli, particularly emotional faces [54, 55]. Functional alterations of the amygdalae appear to characterize especially anxiety-prone [56] or depressed individuals [57, 58], characteristics also displayed by individuals suffering from acute AN [59–61]. As general anxiety levels and negative affect decrease during recovery [62, 63] they might be partly responsible for the fact that the amygdala does not display exaggerated reactivity towards negative emotional stimuli in recAN. Although we did not observe a direct association between depressive or anxiety symptoms and amygdala activity in acAN [20], reported symptoms were only based on self-report data and more objective measures might yield different results [64].

PFC areas have been shown to exert top-down regulatory control over emotion modulating limbic brain regions (such as the amygdala) in order to promote successful emotion regulation [65]. We, therefore, suggested that increased dlPFC activity in acAN may reflect a compensatory response to elevated amygdala reactivity. However, based on assumptions of limited control capacities [66, 67], the opposite could also have been the case. The observed dlPFC hyperactivity could have depleted resources which in turn led to increased vulnerability of the amygdala and increased activity to negative stimuli [68, 69]. This latter interpretation was further supported by our follow-up study [27] that found increased dlPFC activity in acAN patients during the emotion regulation task predicted subsequent higher amygdala response to negative and neutral stimuli that were presented shortly after the main experiment. Given that it might no longer be necessary to constantly regulate (e.g., feelings of hunger and bodily signals promoting eating [62]) after weight-recovery, a speculative interpretation of our current findings in recAN could be that control mechanisms exerted by the dlPFC are only active when needed. Hence, resources needed for regulation (e.g., in the event of negative emotions) might be more readily available following recovery and therefore would not promote increased vulnerability or amygdala reactivity. Our result indicative of equivalent neural activation between recAN and HC is consistent with work on other emotional disorder-unrelated stimuli, which found intact neural processing of emotional stimuli in recAN [36, 70]. However, the results deviate from other neuroimaging studies in recAN that show blunted amygdala activity in response to disorder-unrelated (facial) stimuli [71] or have employed disorder-related stimuli such as food pictures [72–74], taste [75–78], or body stimuli [79].

Although no neural differences in processing and regulation of positive stimuli were observed in our previous study of acAN [21], we did find those patients, who were better able to downregulate neural activity in the VS, were also the ones suffering from worse consequences in everyday life in terms of negative affect, more tension, increased disorder-related rumination and even less weight gain after 60 and 90 days of specialized treatment. Drawing on theories of limited cognitive resources [66], we interpreted worse short- and long-term outcomes as real-life costs of control in terms of possible consequences of maladaptive control tendencies that are common in acAN [29] and an emerging therapeutic target [80, 81]. Relatedly, ironic processing theory [82, 83] states that suppressed thought actually becomes more accessible and backfire with intrusive thoughts (e.g., disorder-related rumination), increased risk of depression, and negative affect. Based on these interpretations, we speculate that control tendencies are either not as strong or not as costly for recAN compared to during the acutely underweight state. This apparent reduction of overcontrol in recAN dovetails with recent findings from our group suggesting that the neural mechanisms underlying self-controlled choice normalize over the course of recovery [70, 84]. Alternatively, it is possible that tendencies to avoid (e.g., suppress) emotions or specific thought content decrease in recAN [34], allowing for more adaptive processing of emotional as well as eating disorder-related content [62] with less intrusive thoughts.

Our findings of similar emotion regulation processes in recAN and HC are in line with questionnaire data reported who also found no differences in self-reported emotion regulation capacities of remitted AN individuals [34, 85]. However, others reported that difficulties with emotion regulation as assessed via self-report persist even after recovery [31], highlighting the importance of applying measures that go beyond questionnaire data.

The following limitations should be considered when interpreting the results of this study: Given the systematic age differences between acAN from our previous manuscript and the weight-recovered sample, we abstained from comparing all three groups in one statistical model which may have delivered different results. Our study was intentionally designed in this manner with the purpose of conducting separate (but nonetheless identical) analyses addressing state vs. trait effects. To clarify the question of normalization of emotional processes during recovery, beyond correlational analysis, future research applying longitudinal study designs is strongly encouraged. Secondly, although we gave explicit instructions including pre-scan training, we cannot be absolutely sure which emotion regulation strategy participants applied during the task. Lastly, in order to avoid anxious emotional states, we only investigated emotion regulation to a stimulus set, which was strictly filtered regarding ED-related content, and therefore our findings may not generalize to disorder-relevant stimulus content [86].

Our previously observed alterations in neural processing of negative emotions, as well as associations between emotion regulation of positive stimuli and outcome measures in acAN [20, 21]) were not visible in recAN individuals. Thus, the current findings provide evidence for a relative normalization following weight-recovery, suggesting that aberrant neural mechanisms underlying emotion processing and regulation might represent state factors associated with undernutrition rather than a disorder-defining trait. Nevertheless, the extent and speed of normalization over the course of recovery remains an open question. Overall, our results may send a positive message to patients, while also being useful information for patient education and therapeutic interventions [81, 87, 88].

## Supplementary information

Supplementary Material
