# Supplementary material for: Intact neural and behavioral correlates of emotion processing and regulation in weight-recovered anorexia nervosa: a combined fMRI and EMA study
Source: Transl Psychiatry. 2022 Jan 24;12:32. doi: 10.1038/s41398-022-01797-1 (PMC8786843; doi:10.1038/s41398-022-01797-1)
Supplement: Supplementary file 1 — Supplementary Material [file 41398_2022_1797_MOESM1_ESM.docx]

**Seidel et al. Intact neural and behavioral correlates of emotion processing and regulation in weight-recovered anorexia nervosa: a combined fMRI and EMA study**

**Supplementary Material**

- 1. Participants

Participants of both the recovered anorexia nervosa (recAN) and healthy control (HC) groups at the focus of the current analyses were excluded if they had a history of organic brain syndrome, schizophrenia, substance dependence, psychosis NOS, bipolar disorder, bulimia nervosa or binge-eating disorder. Further exclusion criteria included IQ<85, psychotropic medication within 6 weeks prior to the study (other than SSRI, n=4), current substance abuse, inflammatory, neurologic or metabolic illness, chronic medical or neurological illness that could affect appetite, eating behavior, or body weight, clinically relevant anemia, pregnancy or breast feeding.

Regarding lifetime psychiatric comorbidities 10 individuals recovered from AN reported having been in psychotherapeutic treatment for other psychiatric disorders than the eating disorder. Six recAN (14.6%) reported having had a depressive episode, three (7.2%) anxiety disorder, one (2.4%) obsessive compulsive disorder.

In attempt to a priori gauge sample sizes necessary for detecting group differences in neural emotion processing and regulation we had conducted a power analyses on the basis of our previously published group differences in emotion processing in acutely ill patients suffering from AN (1). Assuming an effect size in AN relative to HC that is comparable to that observed in the aforementioned study (Cohen's d=0.76-0.91) and an alpha-error probability of 5% (and a power of 80%), we would need a minimum of n=34 participants in each group.

- 1. Material and Procedure

During fMRI, participants were cued to either passively view a set of negative, positive and neutral pictures or to actively downregulate any emotions arising in response to the negative and positive pictures through distancing. Negative, positive and neutral stimuli were selected from the International Affective Picture System (IAPS (2) and the emotional pictures set (Emopics; (3)). The stimuli set excluded AN-relevant images such as pictures of food, pictures that focused on female bodies or sports that are primarily executed to burn calories. Neutral images: 116*, 124*, 126*, 127*, 135*, 140*, 2102, 2514, 2749, 850, 2880, 5020, 5300, 5410, 5720, 7150, 7211, 7491, 7550, 7595. Negative images: 210*, 211*, 213*, 216*, 218*, 219*, 224*, 225*, 255*, 321*, 322*, 325*, 328*, 329*, 1050, 1111, 1220, 1280, 1300, 1930, 2141, 2205, 2683, 2703, 2900, 3280, 3500, 5971, 6212, 6230, 6313, 6550, 6571, 9050, 9290, 9421, 9440, 9630, 9830, 9911. Positive Pictures: 1*, 2*, 3*, 4*, 5*, 6*, 8*, 9*, 66*, 258*, 262*, 1440, 1460, 1710, 1750, 1920, 1999, 2040, 2070, 2340, 2341, 2345, 2360, 2540, 8021, 8030, 8031, 8034, 8120, 8330, 8380, 8490, 8500, 8501. 8502, 8503, 8510, 8540.

All stimuli were presented onto a back-projection screen located at the rear end of the scanner and were viewed through a mirror attached to the head coil. During the view condition participants were instructed to simply view the picture without modulating any associated feelings while not looking away or otherwise distracting themselves in any way. During the regulation condition they were told to try to downregulate any elicited feeling via the reappraisal strategy “distancing”. More specifically they were instructed to: ’Look at the following picture directly, but try to take the position of a noninvolved observer, thinking about the present picture in a neutral way/Imagine that between you and the picture is a wall of glass/Imagine the picture is getting smaller and smaller’.

The instruction for each condition was given by presenting a cue word laid over the stimulus for 1.5 s stating either ‘view’ or ’distance’. Given feasibility and constraints of the current paradigm, the current study adopted the an approach also employed by another group (4) using a combination of early and late cues. We did not include a “distance neutral” condition in the experiment since we assumed no initial emotional reaction that could be downregulated. After each picture presentation (6 s), participants were asked to rate how aroused they were at the current moment on a visual analogue scale (3 s), ranging from ‘very aroused’ to ‘not aroused at all’. We did not include valence ratings of the images used in the experiment of the current study. This decision was primarily made on the basis of feasibility (1. not confusing our younger participants with different ratings and 2. time constraints to avoid that participants did not have to lie in the scanner for too long). A similar setup has been implemented and published previously (4,5). The rating was intended to reflect the critical role of the amygdala in arousal regulation (6). Prior to data collection pilot data of 15 AN patients who rated the selected images on both valence and arousal was collected. Kendall’s concordance coefficient was .90 for all negative stimuli as well as .91 for the positive images, when we compared the piloted ratings to those obtained by Libkumen and colleagues (7) as well as Wessa et al. (3). Thus, our ratings were highly similar to those obtained in those previous studies. The rating was followed by an inter-trial interval that was jittered between 3–5s as randomly drawn from a pseudo-exponential distribution, where 50% of intervals lasted 3s, 25% lasted 3.5s, 12% lasted 4s, 6% lasted 4.5s and 6% lasted 5s, resulting in a mean interval of ~ 3.5s.

To familiarize participants with the procedure and ensure their understanding of the instruction cues prior to entering the MR scanner, they performed a training session which consisted of 17 trials including each condition and lasted approximately 10 minutes. After the practice session, participants were asked if they had any difficulties applying the instructions and to explain how they complied with the regulation instruction. If this report was incompatible with prior instructions or participants reported difficulties with the task, instructions were read again and participants completed another training session. Stimuli used in training were different from those shown in the main experiment.

- 1. Ecological Momentary Assessment

Participants had the choice between a study smartphone (Samsung Galaxy Ace, 5831i) and their own mobile phone.

Participants were initially screened, weighed, and interviewed, before they received detailed instruction on how to handle the smartphone, the MovisensXS-app (MovisensXS, Karlsruhe, Germany) and the content of the questionnaire. They were instructed to answer the questionnaire as soon as the alarm appeared, but were given an additional 30 minutes after the prompt when unable to reply (e.g., during class or work, during meal times or therapy session) or if safety was a concern (e.g., while driving).

EMA sampling started the day after the fMRI scan for a period of 14 days. Recordings during the first days were closely monitored in terms of compliance, to ensure that instructions were understood. Compensation was provided at the end of the study, in accordance with compliance rates.

Data collection was accomplished via the signal-contingent assessment method: Alarms occurred at six semi-random times during a 14 hour period that was adapted for each individual to suit different daily routines. Prompts were anchored within six smaller intervals of two 2 ½ hour intervals (before midday) and for 1 ½ hour intervals (after midday).

- 1. Image Acquisition

Images were acquired between 8 and 9 AM following an overnight fast (to control for the potential influence of acute nutritional intake and/or diurnal hormone rhythms in both groups) with a Siemens 3T MRI scanner (Erlangen, Germany) equipped with a standard head coil. Structural images were acquired with a T1-weighted MPRAGE sequence (TR=1900 ms, TE=2.26 ms, FOV=256×256 mm, 176 slices, 1×1×1 mm3 voxel size, flip angle=9°). For functional imaging, a standard gradient-echo T2*-weighted EPI sequence was used (TR=2410 ms; TE=25 ms; flip angle=80°). A total of 575 volumes were obtained (42 transversal slices orientated 17° clockwise to the AC-PC line, 2 mm slice thickness, 1 mm gap, FOV=192×192 mm, in-plane resolution of 64×64 pixels=voxel size of 3×3×2 mm3). Task presentation and behavioral response recording was performed using Presentation software (Neurobehavioral Systems, Inc., Albany, CA). In addition to standard motion correction during preprocessing, we routinely implement the following quality control procedures in our fMRI data: First, we exclude participants if they show outliers (motion and/or intensity) in more than 25% of all frames (total of 575 in the current time series). This was not the case in the current sample. Secondly, we controlled for volumes that included such outliers with an additional regressor (for each outlier volume) during first level analysis.

- 1. Statistical Analyses
     1. Functional MRI and task-based data

Whole brain results for the contrasts negative watch>neutral, negative watch>negative distance, negative watch<negative distance, positive watch>neutral, positive watch>positive distance, positive watch<positive distance were calculated and visualized at a cluster forming threshold of p<0.001, uncorrected. To calculate group differences between recAN and HC, we applied 2^nd^ level independent samples t-tests. Further, to explore whole brain correlations in the recAN group between negative emotional processing and regulation and rumination (food and weight) and negative affect we calculated three additional GLMs for each contrast (negative watch>neutral, negative watch>negative distance and negative watch<negative distance) and included rumination about food/weight and negative affect separately as a covariate (FWE corrected for false positives).For 2^nd^ level analysis we applied a one sample t-test with one covariate to investigate the effects in the recAN group only.

For the arousal ratings we calculated a positive and negative arousal regulation score (watch -distance for each valence) as in our previous analyses in acute AN (1,8). On the bases of the extracted betas (within amygdala (negative) and VS (positive) masks) we also calculated a positive and negative neural emotion regulation score (subtracted the negative distance from the negative watch condition). To assess whether the neural emotion regulation scores were associated with the arousal regulation scores for each valence, the subscales of the ERQ (reappraisal and suppression) or BMI and BMI-SDS values we used Spearman’s Rho.

- - 1. Hierarchical linear models

To predict EMA measures (a) food rumination and (b) weight rumination, (c) affect or (d) tension, we calculated four different HLMs for each hemisphere (left amygdala, right amygdala). The models were identical as to the ones for our main analysis. For each of the models we allowed for random intercepts and included time (indicating time of day as a continuous variable from 1 to 6) on level 1 and day of study (1 to 14) on level 2. On level 3, the person level, we included three predictors. First diagnostic group was inserted, coded as 1(recAN) and -1(HC). Secondly we added the negative neural regulation score of the amygdala (see supplementary material 1.5.1) as well as the respective interaction between negative neural regulation and group, as predictors to the models at the person-level.

**Results**

- 1. Behavioral Task-based Results

As expected the 5x2 repeated measures ANOVA revealed a main effects of task in the arousal ratings, supporting the general validity of the task (F(4,248.63)=151.58, p<0.001). Post-hoc tests confirmed that arousal in the positive watch and negative watch condition were higher than in the neutral watch condition (both p<0.001) and that it decreased during distancing compared to watch for positive as well as negative stimuli (both p<0.001). There were no group differences.

- 1. Functional MRI Task-based Results

For fMRI main effects of task, see Figure S1 (for negative emotional stimuli) and S2 (for positive emotional stimuli). There was a significant increase in activity during the watch conditions in the bilateral amygdala (negative watch>neutral) and in the VS (positive watch>neutral). There was a significant decrease in activity during distancing for positive stimuli in the VS (positive watch>positive distance) and a significant increase in the dlPFC for negative and positive stimuli during regulation (negative watch<negative distance, positive watch<positive distance). No group differences were observed during the neural processing and regulation of positive and negative emotional stimuli in any of the aforementioned contrasts and ROIs, or in the comparisons at whole-brain level. Rumination and negative affect was not correlated with whole-brain neural activity in either contrast (negative watch>neutral, negative watch>negative distance and negative watch<negative distance) in the recAN group when added as covariate to the model.

- 1. Associations between Behavioral and Neural Task Measures

The negative neural regulation score of the left amygdala was positively correlated with the negative arousal regulation score (Table S1). There was no significant association between the positive neural regulation score of the VS and the positive arousal regulation score (Table S1).

**Supplementary Figures**

**Figure S1: Neural Processing of Negative Stimuli**


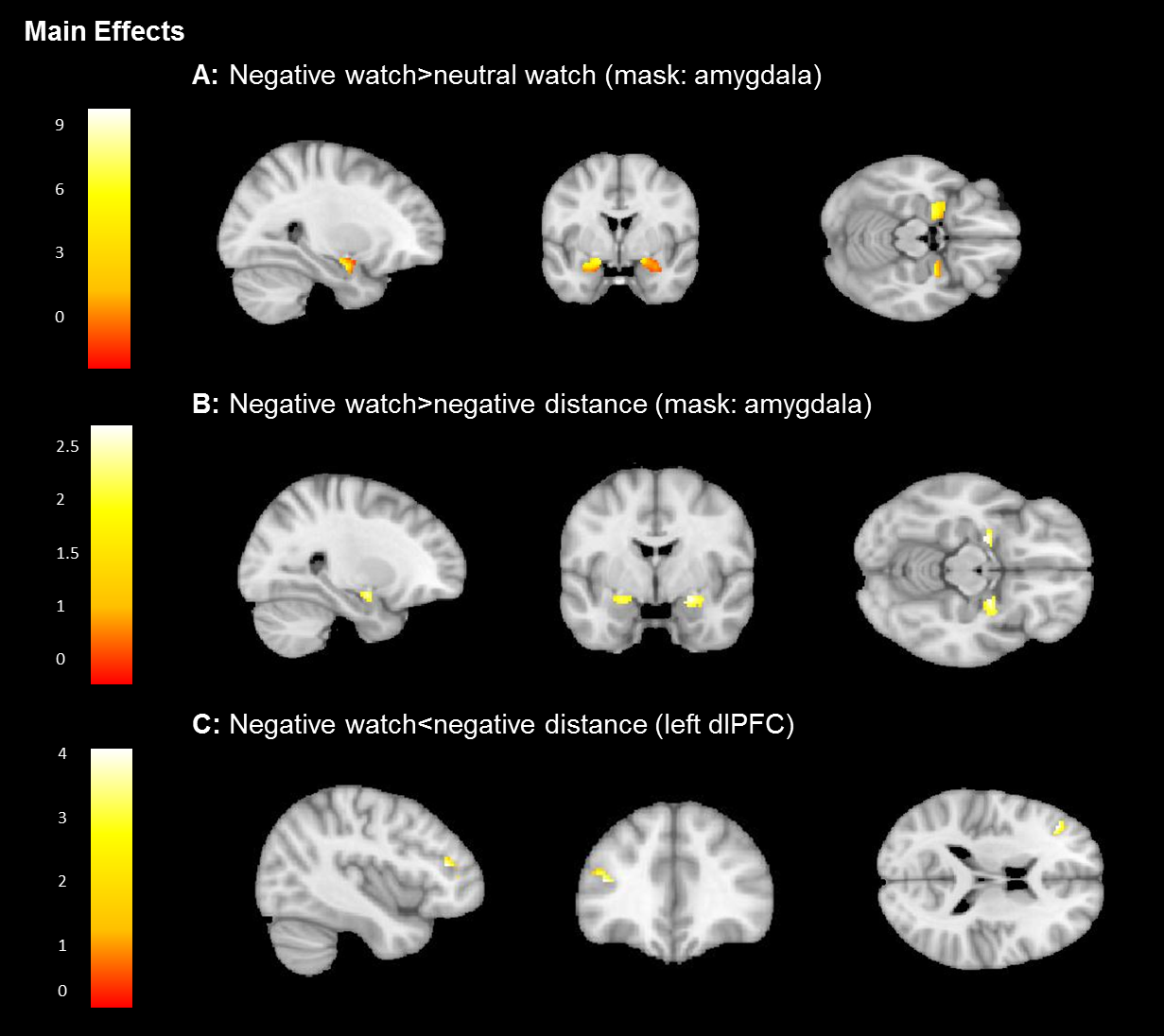


Figure S1: Region of interest (ROI) analysis depicting main effects of task over all participants. ROI were amygdala, and left and right dlPFC. Contrast **A** displays brain activations for passively viewing negative pictures compared to neutral ones (watch>neutral), the amygdala was used as mask. Results are shown at a threshold of p<0.001 uncorrected. As expected watching negative pictures activated large parts of the bilateral amygdala. Contrast **B** displays results of down-regulation (watch>distance) of amygdala during distancing. Results are shown at a threshold of p<0.05 uncorrected (and did not withstand correction for multiple comparisons). Contrast **C** shows results during regulation (watch<distance) of negative emotions using the bilateral dlPFC as mask. Results are shown at a threshold of p<0.001 uncorrected. As expected reappraisal activated this frontal region during effortful regulation. dlPFC=Dorsolateral prefrontal cortex.

**Figure S2: Neural Processing of Positive Stimuli**


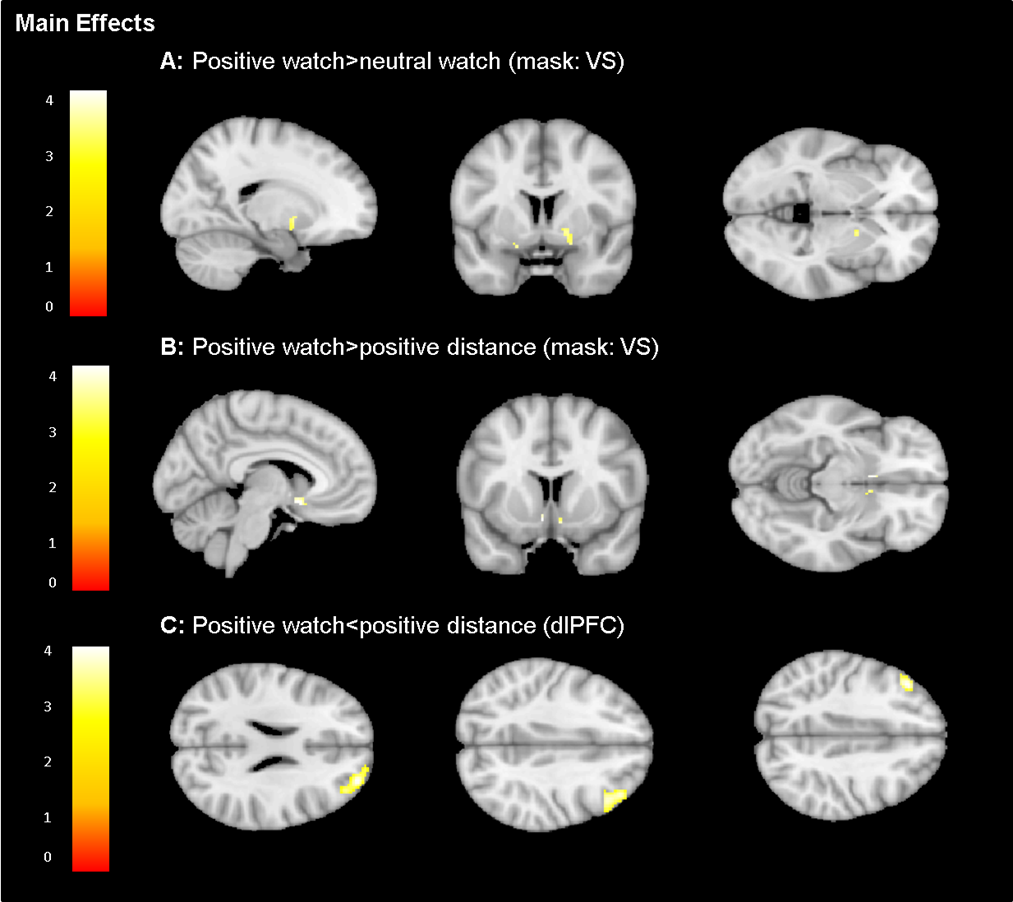


Figure S2: Region of interest (ROI) analysis depicting main effects of task over all participants within the regions of interest (ROIs were left and right VS and left and dlPFC). All results are shown at a threshold of p<0.001 uncorrected. Contrast **A** displays brain activations for passively viewing positive pictures compared to neutral ones (watch>neutral), the VS was used as mask. As expected watching positive stimuli increased activation in the VS. Contrast **B** displays results of down-regulation (watch>distance) of VS during distancing. As expected reappraisal results in decreases of neural responses, indicating that the task was executed successfully. Contrast **C** shows results during effortful regulation (watch<distance) of positive emotions, the bilateral dlPFC was used as mask. As expected reappraisal activated this frontal region during effortful regulation. dlPFC=Dorsolateral prefrontal cortex, VS=Ventral striatum.

**Supplementary Tables**

| **Table S1: Non-parametric Analysis of Descriptive Statistics** | | | | | | | |
| --- | --- | --- | --- | --- | --- | --- | --- |
|  |  | **recAN** | |  | **HC** |  |  |
| **Descriptive Statistics** | **(recAN/HC)** | | **Median** | **IQR** | **Median** | **IQR** |  |
| **BDI-II** | 41/41 | | 9 | 13.5 | 4 | 7.18 | ** |
| **ERQ-Suppression** | 41/41 | | 14 | 8 | 12 | 6 |  |

Descriptive statistics, results of group comparisons using Mann-Whitney-U T-tests, displaying median and interquartile range. **=p<0.01. RecAN=Recovered Anorexia nervosa, HC=Healthy control, BDI-II=Beck depression inventory, ERQ=Emotion regulation questionnaire.

| **Table S2: Association between Arousal and Neural Regulation** | | | | | | | | | | |
| --- | --- | --- | --- | --- | --- | --- | --- | --- | --- | --- |
|  | **Negative** | | | | | | **Positive** | | | |
|  | **Left**  **Amygdala** | **Right**  **Amygdala** | |  | |  | **Left**  **VS** | **Right**  **VS** |  |  |
| **Negative Arousal Regulation** | 0.27* | 0.16 |  | |  | |  |  |  |  |
| **Positive Arousal Regulation** |  |  |  | |  | | -0.09 | -0.06 |  |  |

Correlation coefficients (Spearman’s Rho) between arousal regulation (watch-distance) and neural regulation of extracted betas of regions of interest in the contrast negative watch-negative distance, for positive and negative valence. VS=Ventral striatum, *=p<0.05.

| **Table S3: Correlation Coefficients of Negative Neural Induction Score with BMI and BMI-SDS** | | |  |
| --- | --- | --- | --- |
|  | **BMI (recAN)** | **BMI-SDS (recAN)** | |
| **Amygdala L** | .246 | .238 | |
| **Amygdala R** | .232 | .233 | |
| **dlPFC L** | .042 | .104 | |
| **dlPFC R** | .183 | .152 | |

Spearman’s rho between negative neural induction score with BMI-SDS, recAN=recovered anorexia patients, HC=Healthy controls, BMI=Body-mass-index, BMI-SDS=Body-mass-index standard deviation score, EDI-2-total=Eating Disorder Inventory (total score), BDI-II=Beck Depression Inventory, dlPFC=Dorsolateral prefrontal cortex, L=Left, R=Right. None of the correlations was significant at al alpha-level of p<.05.

| **Table S4: Multilevel estimates for models predicting rumination (food, weight), affect, and tension** | | | | | | | | | | | | | | | |
| --- | --- | --- | --- | --- | --- | --- | --- | --- | --- | --- | --- | --- | --- | --- | --- |
|  | **Food (a)** | |  | | **Weight (b)** | |  | **Affect (c)** | | |  | **Tension (d)** | | | |
| **Parameter** | **Beta** | **SE** | | **p** | **Beta** | **SE** | **p** | **Beta** | **SE** | **p** | | | **Beta** | **SE** | **p** |
| **Fixed effects** |  |  | |  |  |  |  |  |  |  | | |  |  |  |
| **Intercept** | 35.15 | 1.87 | | <.001 | 24.89 | 2.06 | <.001 | 135.17 | 3.43 | <.001 | | | 130.41 | 3.74 | <.001 |
| **Group** | -1.32 | 1.87 | | n.s. | 7.91 | 2.06 | <.001 | -11.24 | 3.43 | 0.002 | | | -9.78 | 3.74 | 0.011 |
| **Neural Regulation VS** | -3.46 | 11.22 | | n.s. | 1.76 | 10.93 | n.s. | 1.76 | 20.26 | n.s | | | -12.26 | 19.41 | n.s. |
| **Neural Regulation VS × Group** | 10.60 | 11.22 | | n.s. | -2.38 | 10.93 | n.s. | 3.16 | 20.26 | n.s. | | | 1.2 | 19.41 | n.s. |

Multilevel estimates for models predicting rumination (food, weight), affect, and tension. SE=Standard error, Group=-1(HC), 1(recAN), Day=Day within study, Time=Prompt within day, Neural regulation score VS=positive watch - positive distance of extracted parameter estimates of ventral striatum.

| **Table S5: Multilevel estimates for models predicting rumination (food, weight), affect, and tension** | | | | | | | | | | | | | | | |
| --- | --- | --- | --- | --- | --- | --- | --- | --- | --- | --- | --- | --- | --- | --- | --- |
|  | **Food (a)** | |  | | **Weight (b)** | |  | **Affect (c)** | | |  | **Tension (d)** | | | |
| **Parameter** | **Beta** | **SE** | | **p** | **Beta** | **SE** | **p** | **Beta** | **SE** | **p** | | | **Beta** | **SE** | **p** |
| **Fixed effects** |  |  | |  |  |  |  |  |  |  | | |  |  |  |
| **Intercept** | 35.37 | 1.86 | | <0.001 | 25.1 | 1.99 | <0.001 | 134.48 | 3.4 | <0.001 | | | 129.53 | 3.61 | <0.001 |
| **Group** | -1.4 | 1.86 | | n.s. | 7.57 | 1.99 | <0.001 | -10.41 | 3.4 | 0.003 | | | -9.12 | 3.61 | 0.014 |
| **Neural Regulation Amygdala right** | 5 | 6.02 | | n.s. | 0.56 | 7.99 | n.s. | 11.52 | 12.34 | n.s. | | | 9.14 | 13.22 | n.s. |
| **Neural Regulation Amygdala right × Group** | 10.01 | 6.02 | | n.s. | 14.81 | 7.99 | n.s | -22.55 | 12.34 | n.s. | | | -26.41 | 13.22 | n.s. |

| **Table S6: Multilevel estimates for models predicting rumination (food, weight), affect, and tension** | | | | | | | | | | | | | | | |
| --- | --- | --- | --- | --- | --- | --- | --- | --- | --- | --- | --- | --- | --- | --- | --- |
|  | **Food (a)** | |  | | **Weight (b)** | |  | **Affect (c)** | | |  | **Tension (d)** | | | |
| **Parameter** | **Beta** | **SE** | | **p** | **Beta** | **SE** | **p** | **Beta** | **SE** | **p** | | | **Beta** | **SE** | **p** |
| **Fixed effects** |  |  | |  |  |  |  |  |  |  | | |  |  |  |
| **Intercept** | 35.23 | 1.89 | | <0.001 | 24.77 | 2.04 | <0.001 | 134.09 | 3.58 | <0.001 | | | 129.48 | 3.54 | <0.001 |
| **Group** | -1.25 | 1.89 | | n.s. | 7.82 | 2.04 | <0.001 | -10.4 | 3.58 | 0.005 | | | -9.16 | 3.54 | 0.012 |
| **Neural Regulation Amygdala left** | 4.85 | 5.22 | | n.s. | 5.91 | 6.47 | n.s. | 12.94 | 10.32 | n.s. | | | 7.90 | 10.73 | n.s. |
| **Neural Regulation Amygdala left × Group** | 3.75 | 5.22 | | n.s. | 7.22 | 6.47 | n.s. | -16.17 | 10.32 | n.s. | | | -17.93 | 10.73 | n.s. |

Multilevel estimates for models predicting rumination (food, weight), affect, and tension. SE=Standard error, Group=-1(HC), 1(recAN), Day=Day within study, Time=Prompt within day, Neural regulation score Amygdala right=Negative watch - negative distance of extracted parameter estimates of right amygdala.

Multilevel estimates for models predicting rumination (food, weight), affect, and tension. SE=Standard error, Group=-1(HC), 1(recAN), Day=Day within study, Time=Prompt within day, Neural regulation score Amygdala left=Negative watch - negative distance of extracted parameter estimates of left amygdala.

**References**

1. Seidel M, King JA, Ritschel F, Boehm I, Geisler D, Bernardoni F, *et al.* (2018): Processing and regulation of negative emotions in anorexia nervosa: An fMRI study. *NeuroImage Clin* 18: 1–8.

2. Lang P, Bradley M, Cuthbert B (2008): *International Affective Picture System (IAPS): Affective Ratings of Pictures and Instruction Manual*.

3. Wessa M, Kanske, P, Neumeister P, Bode K, Heissler J, Schönfelder S (2010): EmoPicS: Subjektive und psychophysiologische Evaluation neuen Bildmaterials für die klinisch-biopsychologische Forschung.

4. Diers K, Weber F, Brocke B, Strobel A, Schönfeld S (2014): Instructions matter: a comparison of baseline conditions for cognitive emotion regulation paradigms. *Front Psychol* 5.

5. Scheffel C, Diers K, Schönfeld S, Brocke B, Strobel A, Dörfel D (2019): Cognitive emotion regulation and personality: an analysis of individual differences in the neural and behavioral correlates of successful reappraisal. *Personal Neurosci* 2. https://doi.org/10.1017/pen.2019.11

6. Hamann S (2012): Mapping discrete and dimensional emotions onto the brain: controversies and consensus. *Trends Cogn Sci* 16: 458–466.

7. Libkuman TM, Otani H, Kern R, Viger SG, Novak N (2007): Multidimensional normative ratings for the International Affective Picture System. *Behav Res Methods* 39: 326–334.

8. Seidel M, King JA, Ritschel F, Boehm I, Geisler D, Bernardoni F, *et al.* (2018): The real-life costs of emotion regulation in anorexia nervosa: a combined ecological momentary assessment and fMRI study. *Transl Psychiatry* 8: 28.
